# Supplementary material for: Fabrication-induced even-odd discrepancy of magnetotransport in few-layer MnBi2Te4
Source: Nat Commun. 2024 Apr 22;15:3399. doi: 10.1038/s41467-024-47779-3 (PMC11035656; doi:10.1038/s41467-024-47779-3)
Supplement: Supplementary file 1 — Supplementary Information [file 41467_2024_47779_MOESM1_ESM.pdf]

Supplementary Information

Fabrication-induced even-odd discrepancy of magnetotransport in few-layer $MnBi_2Te_4$

Yaoxin Li[1,*], Yongchao Wang[1,*], Zichen Lian[1,*], Hao Li[2,3], Zhiting Gao[4], Liangcai Xu[1], Huan Wang[5,6], Rui'e Lu[7], Longfei Li[8], Yang Feng[6], Jinjiang Zhu[9], Liangyang Liu[1], Yongqian Wang[5,6], Bohan Fu[5,6], Shuai Yang[5,6], Luyi Yang[1,10], Yihua Wang[9,11], Tianlong Xia[5,6], Chang Liu[12], Shuang Jia[8,13,14], Yang Wu[15], Jinsong Zhang[1,10,16], Yayu Wang[1,10,16], Chang Liu[5,6†]

[1]State Key Laboratory of Low Dimensional Quantum Physics, Department of Physics, Tsinghua University, Beijing 100084, China

[2]School of Materials Science and Engineering, Tsinghua University, Beijing 100084, China

[3]Tsinghua-Foxconn Nanotechnology Research Center, Department of Physics, Tsinghua University, Beijing 100084, China

[4]Beijing Academy of Quantum Information Sciences, Beijing 100193, China

[5]Beijing Key Laboratory of Opto-electronic Functional Materials & Micro-Nano Devices, Department of Physics, Renmin University of China, 100872 Beijing, China

[6]Key Laboratory of Quantum State Construction and Manipulation (Ministry of Education), Renmin University of China, Beijing 100872, China

[7]School of Mechanical and Electric Engineering, Guangzhou University, Guangzhou 510006, China

[8]International Center for Quantum Materials, School of Physics, Peking University, Beijing 100871, China

[9]State Key Laboratory of Surface Physics and Department of Physics, Fudan University, Shanghai 200433, China

[10]Frontier Science Center for Quantum Information, Beijing 100084, China

[11]Shanghai Research Center for Quantum Sciences, Shanghai 201315, China.

[12]Shenzhen Institute for Quantum Science and Engineering and Department of Physics, Southern University of Science and Technology, Shenzhen 518055, China

[13]Interdisciplinary Institute of Light-Element Quantum Materials and Research Center for Light-Element Advanced Materials, Peking University, Beijing 100871, China

[14]CAS Center for Excellence in Topological Quantum Computation, University of Chinese Academy of Sciences, Beijing 100190, China

[15]College of Math and Physics, Beijing University of Chemical Technology, Beijing 100029, China

[16]Hefei National Laboratory, Hefei 230088, China

* These authors contributed equally to this work.

† Emails: liuchang_phy@ruc.edu.cn

Contents:

A: Fabrication and characterization of few-layer $MnBi_2Te_4$ devices

B: Temperature dependent transport properties for device S2 and S6 at the CNPs

C: Colormap of transport data in device S2 and S6

D: Optical contrast variation after undergoing different fabrication process

E: Atomic force measurements in devices before and after fabrication

F: MOKE variation in two samples before and after contact with PMMA

G: Optical contrast and transport results in samples fabricated by the modified method

H: V_g dependent nonlocal transport behaviors in device S6 and S7

Figure S1 to S11

A: Fabrication and characterization of few-layer $MnBi_2Te_4$ devices

Few-layer $MnBi_2Te_4$ were cleaved onto the SiO_2/Si substrates pre-cleaned by air plasma via Scotch tapes (Step 1 and Step 2). The step-by-step fabrication process is displayed in Fig. S1. After mechanically scratching off the redundant parts around the target flake with a needle (Step 3) in an argon-filled glove box, an optical image of the flakes was taken and the O_c was calculated according to the RGB-values of the flakes and the substate. Next, a 270 nm PMMA layer was spin-coated on the surface and heated to 60 °C for 5 minutes in the glove box before EBL (Step 4). After patterning Hall bar structures by a standard EBL method (Step 5), Cr/Au electrodes were deposited through thermal evaporation. The PMMA layer was then lifted off by acetone. Before transport measurements, a 270 nm thick PMMA layer was spin-coated following the same procedure as Step 4 for protection.

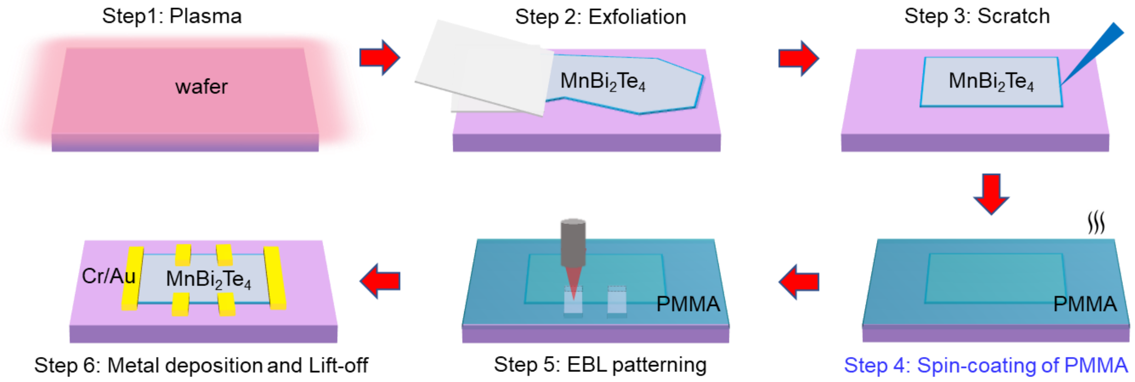

Fig. S1 | Schematics of the fabrication process of $MnBi_2Te_4$ Hall bar devices. Step 1, Air-plasma cleaning of SiO_2/Si substrate. **Step 2,** Exfoliating $MnBi_2Te_4$ flakes by Scotch tapes. **Step 3,** Removing redundant part with a needle and identifying the thickness by a microscope. **Step 4,** Spin-coating of 270 nm thick PMMA before EBL. **Step 5,** Standard EBL process for patterning Hall bar structure. **Step 6,** Deposition of Cr/Au electrodes by thermal evaporation.

In order to determine the thickness of the $MnBi_2Te_4$ thin flakes and its relation with O_c, we performed atomic force microscopy measurements on several samples. Results from three typical samples are displayed in Fig. S2. For the flakes with O_c of 10.9 %, 10.7 % and 13.5 %, atomic force microscopy measurements suggest that the step heights are 9.4 nm, 9.2 nm, and 9.2 nm, which correspond to the layer number of 7 SLs. For the flakes with O_c of 29.5 % and 42.8 %, the step heights are 10.5 nm and 11.9 nm, respectively, which correspond to the layer

number of 8 SLs and 9 SLs. These results all confirm the relation between O_c and the sample thickness shown in the main Fig. 1d, and agree well with the result in a previous report[1].

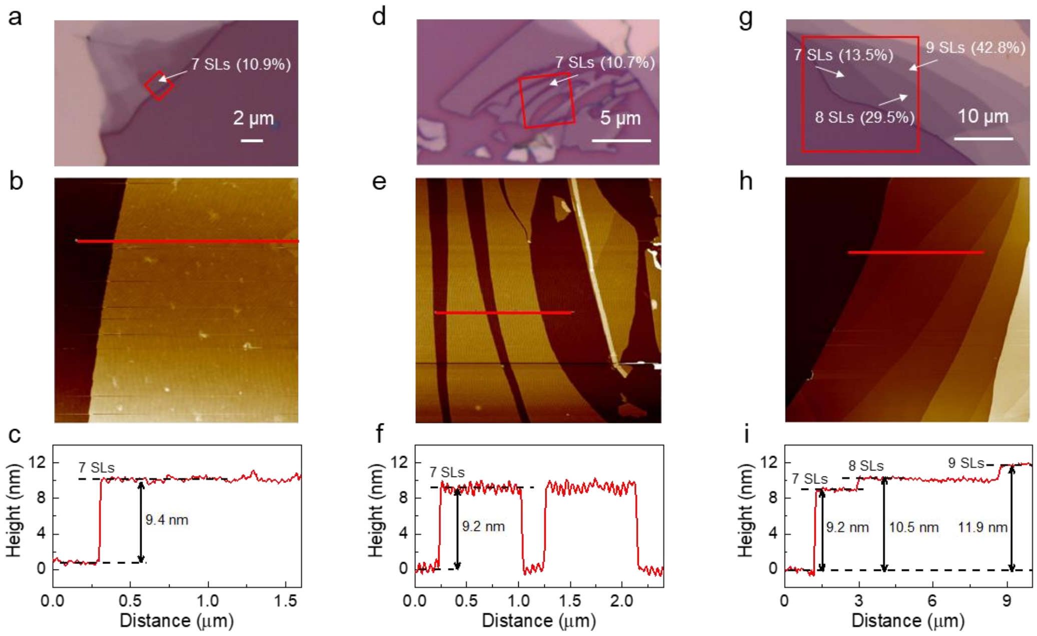

Fig. S2 | O_c and atomic force microscopy measurements of $MnBi_2Te_4$ thin flakes. a, **d**, **g**, Optical images of typical few-layer $MnBi_2Te_4$ flakes exfoliated on SiO_2/Si substrates. **b**, **e**, **h**, Atomic force microscopy measurements of the areas marked by red boxes in optical images. **c**, **f**, **i**, Cross-sectional profiles of the steps along the red lines.

To substantiate the relation between O_c and thickness, we performed a DC magnetic flux measurement on two devices exhibiting various thickness by scanning SQUID. The scanning SQUID probe is very sensitive for detecting magnetic flux from magnetic domains, therefore is highly valuable for checking the calibration of thickness in 2D magnets. Both two samples were exfoliated from crystal #1, which is the least sensitive to fabrication in all our 4 crystals. Figure S3 shows the optical images and the DC flux imaging results for the two devices. For the $MnBi_2Te_4$ sample with $O_c \sim 13.4$ %, its thickness corresponds to 7 SLs. In alignment with expectations, the DC flux imaging revealed a pronounced magnetization when $\mu_0 H$ was swept to zero from positive (Fig. S3b). For the second device, due to the varied thickness across the sample, the contrast of the optical properties and the DC flux are much clearer. In the region where O_c is around -20.1 %, which corresponds to the thickness of 5 SLs, a net magnetization was observed in the scanning SQUID measurement. Conversely, in the part with O_c of -1.3 %,

which corresponds to the thickness of 6 SLs, the magnetization is much smaller. These results confirm the accuracy of our thickness calibration. More detailed information concerning the 5- and 6-SL sample can be refereed to reference[2].

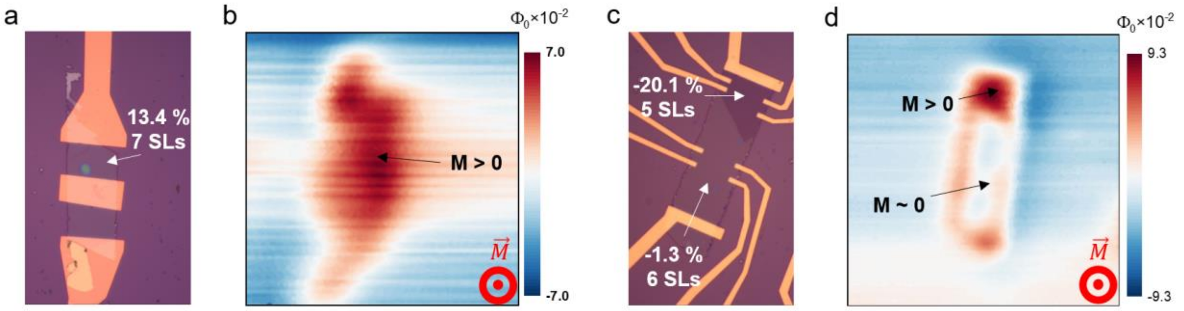

Fig. S3 | Optical images and scanning SQUID results in $MnBi_2Te_4$ with varied thickness. **a,** Optical image of a 7-SL $MnBi_2Te_4$. **b,** DC magnetic flux imaging of the sample by scanning SQUID when μ_0H was swept to zero from positive direction. **c,** Optical image of a $MnBi_2Te_4$ device with two thicknesses. **d,** DC magnetic flux imaging of the device across different parts of the sample. **d** is adapted from reference 2.

B: Temperature dependent transport properties for device S2 and S6 at the CNPs

We probed the magnetotransport properties of the two 6- and 7-SL devices (S2 and S6) shown in the main text at different Ts. Figure S4a displays the μ_0H dependent ρ_{yx} and ρ_{xx} at various Ts for the 6-SL device. At high Ts, ρ_{yx} shows an overall negative slope with increasing μ_0H. With the decrease of T, a wide zero Hall plateau forms at low field regime. Meanwhile, ρ_{xx} exhibits an insulating behavior and increases to 4 h/e^2 at T = 1.5 K. Such behaviors are consistent with the axion insulator state in 6-SL $MnBi_2Te_4$ (ref. [3-5]). When an out-of-plane μ_0H polarizes $MnBi_2Te_4$ into FM order, the system enters the Chern insulator characterized by dissipationless chiral edge states. At μ_0H = 8 T, ρ_{yx} is almost quantized (0.996 h/e^2) and ρ_{xx} drops to a small value (0.002 h/e^2). Generally, the zero Hall plateau is specific for even-number-SL $MnBi_2Te_4$ with preserved time-space (PT) symmetry and should not be observed in odd-number-SL $MnBi_2Te$ with uncompensated AFM order. However, we unexpectedly observed a zero Hall plateau in the 7-SL device, though less perfected than that of the 6-SL device. The μ_0H dependent ρ_{yx} and ρ_{xx} at varied Ts of the 7-SL device is presented in Fig.

S4b. With decreasing T, ρ_{yx} evolves to three plateaus in $\pm h/e^2$ and 0. Meanwhile, ρ_{xx} increases to 1.5 h/e^2 at low $\mu_0 H$ regime and drops to 0.03 h/e^2 at high $\mu_0 H$ regime.

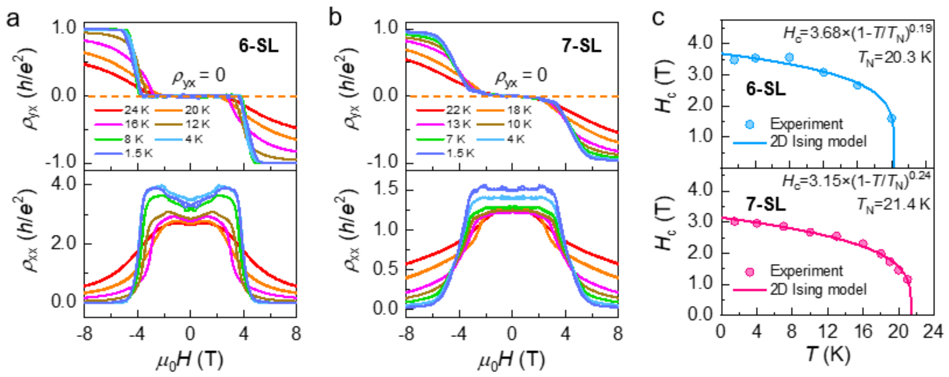

Fig. S4 | Magnetoelectric transport properties of 6- and 7-SL $MnBi_2Te_4$ devices at varied *T*s. a, b, $\mu_0 H$ dependent ρ_{xx} and ρ_{yx} at varied Ts for 6-SL (**a**) and 7-SL (**b**) $MnBi_2Te_4$. $\mu_0 H$ is swept back and forth between -8 T and +8 T. No hysteresis is observed near zero $\mu_0 H$ regime. The orange dashed lines at $\rho_{yx} = 0$ is added for guiding the zero Hall plateau in the AFM state. **c**, Critical field H_c as a function of T for the two devices. The solid circles are the data points extracted from the transport measurements, and the blue and red lines denote the data fittings in the form of $(1\text{-}T/T_N)^{\beta}$.

In an A-type layered antiferromagnet, the onset magnetic field H_c for plateau transition can be regarded as the exchange bias field exerted to the flipping layer by others, as revealed in the study of a prototype A-type van der Waals antiferromagnet CrI_3 (ref. [6]). Therefore, the evolution of H_c as T provides a way to access the thickness dependent magnetic properties of the two devices. Following the same strategy, we extracted the values of H_c and plotted them as a function of T in Fig. S4c. The blue and red circles represent the extracted data and the solid lines are the fittings using $H_c \sim (1\text{-}T/T_N)^{\beta}$, where β is the critical exponent[7]. The extracted T_Ns for the 6- and 7-SL device are 20.3 and 21.4 K, respectively, which are in good agreement with that measured from the R-T curves in Fig. 1e. The critical exponents of the 6- and 7-SL devices are $\beta \sim 0.19$ and 0.24, respectively, which is smaller than $\beta \sim 0.32$ for $MnBi_2Te_4$ bulk crystals[8,9]. This is consistent with the expectation of the crossover from 3D to 2D Ising type magnetism with decreasing thickness[10]. Because neither the 6- nor 7-SL $MnBi_2Te_4$ is in the extreme 2D limit, their β values are still larger than 1/8 derived from 2D Ising model[7].

C: Colormap of transport data in device S2 and S6

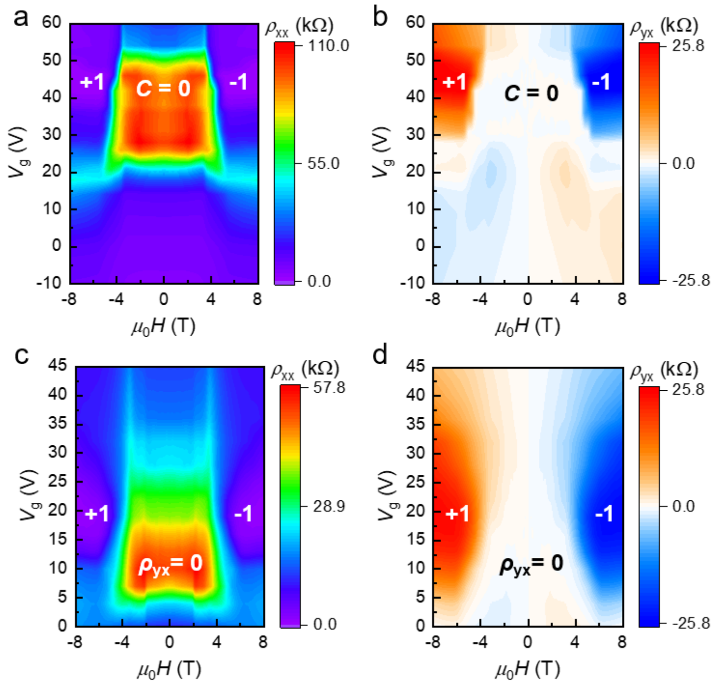

Fig. S5 | Contour plots of experimental data for 6- and 7-SL devices. a, b, Experimental phase diagrams of ρ_{xx} (**a**) and ρ_{yx} (**b**) in the $\mu_0 H$ and V_g plane for the 6-SL device S2 (**c**) and the 7-SL device S6 (**d**).

Figure S5 displays the experimental phase diagrams of the 6-SL device S2 and the 7-SL device S6, which are derived from the $\mu_0 H$ dependent ρ_{xx} and ρ_{yx} at varied V_gs. For the 6-SL device, starting from V_g = -10 V, hole-type carriers are gradually depleted with increasing V_g. At V_g = 46 V, the axion insulator phase with zero Hall plateau appears in the low $\mu_0 H$ regime, as indicated by the orange and white in Figs. S5a and S5b. In the same V_g regime, increasing $\mu_0 H$ drives the AFM state to the field-polarized FM state, and the Chern insulator phase ($C = \pm 1$) with quantized ρ_{yx} and vanished ρ_{xx} appears. All the results are highly consistent with our previous report on the realization of the axion insulator and the Chern insulator phase in a 6-SL $MnBi_2Te_4$ device[3].

For the 7-SL device, despite the presence of a similar zero Hall plateau at some specific V_gs, the colormaps exhibit quantitatively different features. The most pronounced distinction lies in that the zero Hall plateau in the low-field-regime and the Chern insulator in high-field-

regime do not coincide in the same V_g range. For the zero Hall plateau, it appears at $V_g \sim 13$ V, whereas the Chern insulator starts to form at 20 V. The second difference lies in the range of V_g for the zero Hall plateau. For the 6-SL device, the zero Hall plateau is highly robust and survives in a broad V_g range over 10 V. However, for the 7-SL device, it only lives in a narrow V_g regime. These discrepancies strongly indicate that the zero Hall plateau in 6-SL $MnBi_2Te_4$ is indeed a gapped quantized Hall state, while the zero Hall phenomenon in the 7-SL device is likely to be accidental.

D: Optical contrast variation after undergoing different fabrication process

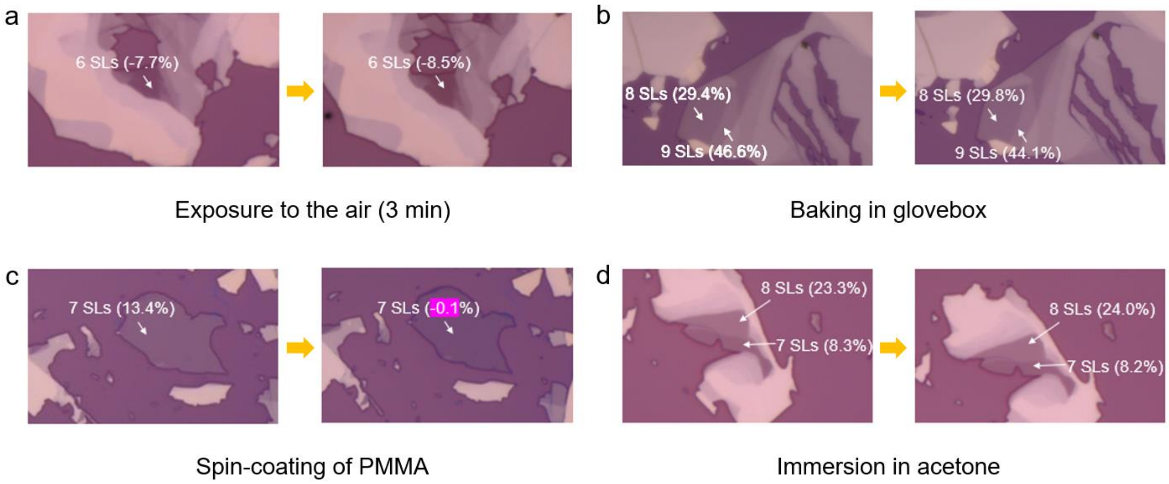

Fig. S6 | O_c variation of four devices after undergoing different fabrication steps. Optical images of $MnBi_2Te_4$ flakes and their O_c variations after being exposed to the air for 3 minutes (**a**), being heated in the glove box at 60 °C for 5 minutes (**b**), being in contact with PMMA for 5 minutes in the glove box (**c**), and immersion in acetone for 15 minutes (**d**).

In order to determine the exact factors that affect the O_c of $MnBi_2Te_4$ flakes, we compare the O_c values of various $MnBi_2Te_4$ flakes under different fabrication conditions. To maximize the experimental effect, we studied the O_c values in the flakes derived from the most sensitive crystal (#4). By directly comparing the color change after different fabrication steps, one can easily identify which factor plays the most significant role on the O_c of $MnBi_2Te_4$. As shown in Fig. S6, these optical pictures clearly show that being exposed to the air, being heated in the glove box and immersion in acetone makes no difference to O_c values. In sharp contrast,

for the flake undergoing a direct contact with PMMA, its O_c value is greatly reduced (see Fig. S6c). These experiments demonstrate that the contact with PMMA has the greatest impact on the O_c value of $MnBi_2Te_4$ flakes.

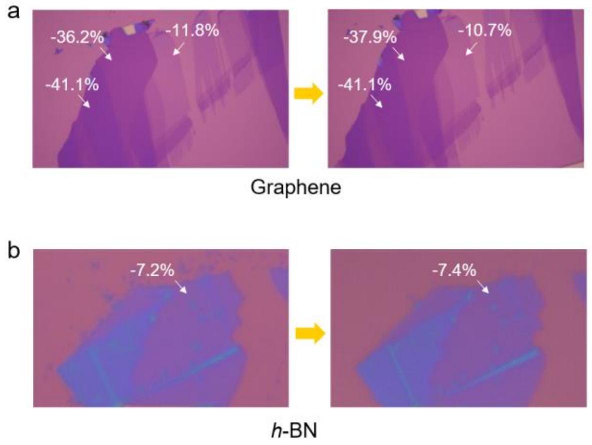

Fig. S7 | Influence of PMMA on O_c in graphene and *h*-BN. Optical images of graphene (**a**) and *h*-BN (**b**) before (left) and after (right) being in contact with PMMA.

To further demonstrate the O_c change arises from the $MnBi_2Te_4$, we performed a contrast experiment on the influences of PMMA on two commonly used 2D materials, graphene, and *h*-BN. As clearly displayed in Fig. S7, neither graphene nor *h*-BN exhibits O_c change after PMMA contact under the same parameter. Therefore, one could conclude that the O_c change due to PMMA is specific for $MnBi_2Te_4$.

E: Atomic force measurements in devices before and after fabrication

In Fig. S8, we present the optical images and the atomic force microscopy measurements results of $MnBi_2Te_4$ flakes before and after being in contact with PMMA. The optical images clearly show that PMMA has a significant impact on the color of $MnBi_2Te_4$ flakes. All these flakes, regardless of their thicknesses, exhibit pronounced O_c reduction after being in contact with PMMA. Figures S8e and S8f display the atomic force microscopy results on these flakes acquired after mechanical exfoliation and after removal of PMMA. Apart from the increased roughness due to PMMA residuals, the step heights for the samples remain almost unchanged within the accuracy of our measurement. This experiment undoubtedly demonstrates that the O_c reduction in $MnBi_2Te_4$ does not arise from the removal of physical layer.

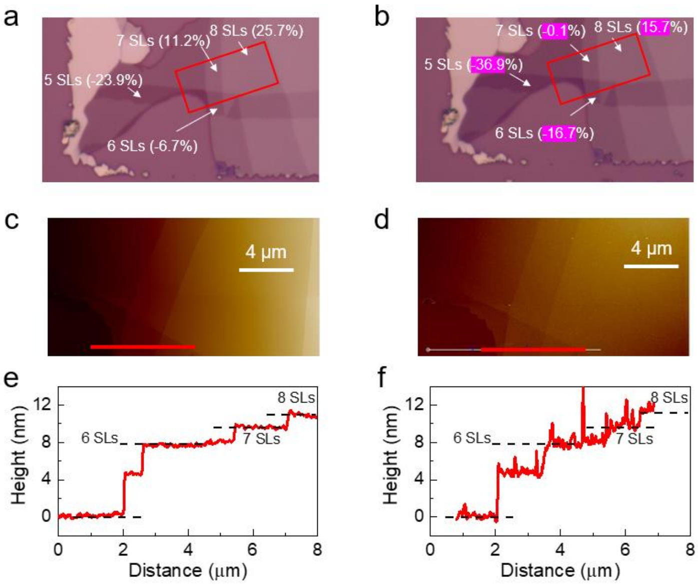

Fig. S8 | Optical images and atomic force microscopy measurements of $MnBi_2Te_4$ flakes after exfoliation and after being in contact with PMMA. a, b, Optical images of $MnBi_2Te_4$ flakes after mechanical exfoliation **a** and after being in contact with PMMA **b**. **c, d,** Atomic force microscopy images of the areas labelled by red boxes. **e, f,** Cross-sectional profiles of $MnBi_2Te_4$ flakes along the red lines in **c** and **d**.

F: MOKE variation in two samples before and after contact with PMMA

In this section, we discussed the results of polar MOKE measurement on two devices of varied thickness. By directly comparing the MOKE variations in the same sample before and after contact with PMMA, we provide further evidence for the influence of fabrication on the effective thickness. As displayed in Fig. S9a, for the sample with an initial O_c of 12.8 %, the thickness corresponds to 7 SLs according to the relation between O_c and thickness. This value was also confirmed by the measurement of coherent interlayer phonon frequency. As shown in Fig. S9b, the coherent interlayer phonon frequency was measured to be around ~ 184 GHz, in line with the value for 7-SL $MnBi_2Te_4$ measured in our previous report[11]. After the PMMA contact, its O_c decreases significantly to 1.8 %, indicating a reduction of effective thickness. Correspondingly, we observed a weakening of magnetization, as marked by the arrow in Fig. S9c. The different variations in the Kerr rotation and Kerr ellipticity signal mainly result from

the different responses of the real and imaginary parts of the optical conductivity to the probe wavelength. However, the coherent interlayer phonon frequency exhibits negligible change after PMMA contact, which suggests that the physical thickness remains essentially unaltered. Reproducible results in another sample with different thickness (5 SLs) are presented in Figs. S9d to S9f. Notably, the entire measurements were carried without a protective layer on the sample surface, therefore, we cannot avoid the potential influence of various extrinsic factors on the sample quality, such as environmental doping, aging effect, and surface oxidation[12-15]. We indeed observed that the initial MOKE signal for both two samples exhibit characteristics deviating from ideal odd-number-SL $MnBi_2Te_4$, suggesting potential damages from extrinsic effects during the exfoliation or transfer process. Nevertheless, these highly consistent results provide compelling evidence for our experiments, validating our calibration of thickness and demonstrating the influence of the fabrication on the effective thickness.

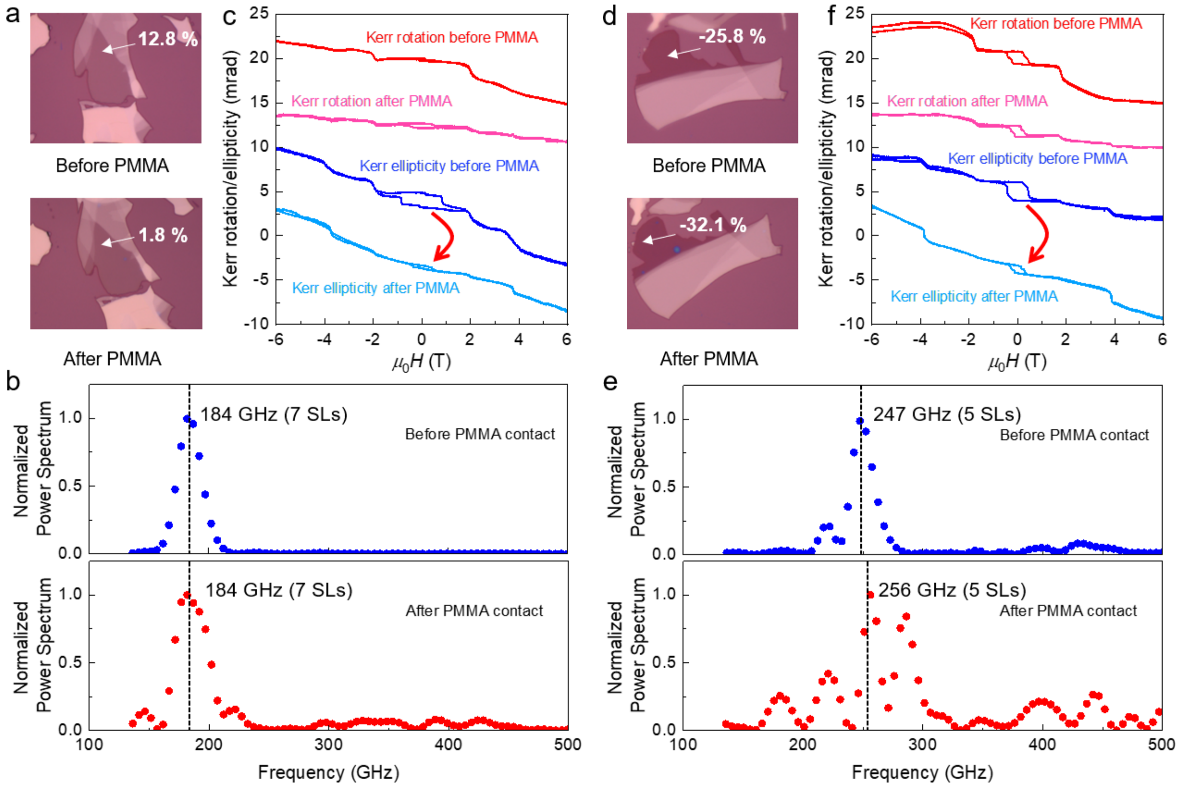

Fig. S9 | Optical images and MOKE measurements of two $MnBi_2Te_4$ samples before and after PMMA contact. a-c, Optical images, MOKE, and phonon frequency results for a 7-SL device before and after PMMA contact. **d-f,** Optical images, MOKE, and phonon frequency results for a 5-SL device before and after PMMA contact.

G: Optical contrast and transport results in samples fabricated by the modified method

To prevent the direct contact of $MnBi_2Te_4$ with PMMA, we thermally deposited a 3-nm AlO_x on the $MnBi_2Te_4$ surface prior to spin-coating process. A mechanic etching process was employed to the capping layer before EBL. This modified fabrication allows us to fabricate $MnBi_2Te_4$ devices without apparent O_c change. In Fig. S10a, we show our preliminary results measured on 22 flakes derived from the same crystal (#5). For the 12 flakes without capping layer, all of them exhibit pronounced O_c changes after fabrication. However, for the other 10 flakes with AlO_x capping layer, their O_c values remain unchanged. This result indicates that AlO_x capping layer can protect the sample from damage caused by PMMA to a large extent. Figure S10b shows the $\mu_0 H$ dependent ρ_{yx} of a 7-SL $MnBi_2Te_4$ with AlO_x capping layer. The anomalous Hall resistivity ~ 0.1 h/e^2 at zero fields has reached the highest value compared to our previous results. More thorough studies are needed to refine the fabrication process.

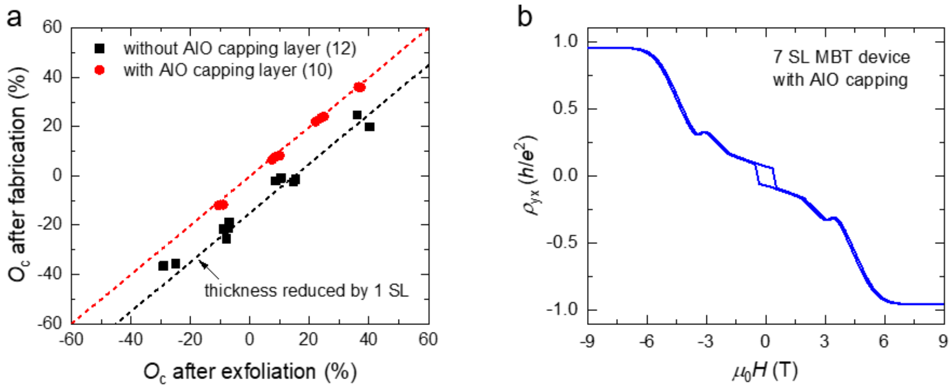

Fig. S10 | Preliminary results of O_c variation and transport in samples fabricated by the modified method. a, Summary of the O_c variation for 22 $MnBi_2Te_4$ flakes with and without AlO_x capping layer. **b**, $\mu_0 H$ dependent ρ_{yx} in a 7-SL device with AlO_x capping layer.

H: V_g dependent nonlocal transport behaviors in device S6 and S7

In this session, we discuss the nonlocal transport behaviors of the “7”-SL and “8”-SL devices (S6 and S7), both of which exhibit apparent O_c reduction after fabrications. Figures S11a and S11b show the different Hall-bar configurations of the local and nonlocal transport

measurements respectively for the two six-terminal devices. We define the local (R_{local}) and nonlocal resistance ($R_{nonlocal}$) as $R_{local} = V_{23}/I_{14}$ and $R_{nonlocal} = V_{34}/I_{16}$. According to previous experiments and theories[4,5], an odd-number-SL device will exhibit a vanishing $R_{nonlocal}/R_{local}$ due to the dissipationless chiral edge state. In contrast, for even-number-SL $MnBi_2Te_4$, a pair of counter-propagating currents is expected to give rise to a large $R_{nonlocal}/R_{local}$ of about 33 % estimated by Landauer–Büttiker formalism. These expected numbers for ideal odd- and even-number-SL devices are marked by the orange and blue lines in Figs. S11d and S11f. However, if there is a mechanism that reduces the effective thickness, the nonlocal behaviors would be totally different.

Figures S11c and S11d show the results of local and nonlocal measurements for the two devices (S6 and S7). For a better comparison between the nonlocal results and the theoretical expectations, we plot the ratio of $R_{nonlocal}$ and R_{local} as a function of V_g. Interestingly, both two devices display apparent deviations from the theoretical expectations. For the "7"-SL device, the $R_{nonlocal}/R_{local}$ signal shows a significant enhancement from 0 to 20 % at the CNP, in sharp contrast to the expected vanishing nonlocal signal expected for odd-number-SL devices. On the contrary, the "8"-SL device S7 exhibits a pronounced suppression of $R_{nonlocal}/R_{local}$ signal from 33 % to 7 %. These results again demonstrate that the effective thickness for transport in samples with severe O_c change is different from their nominal thickness.

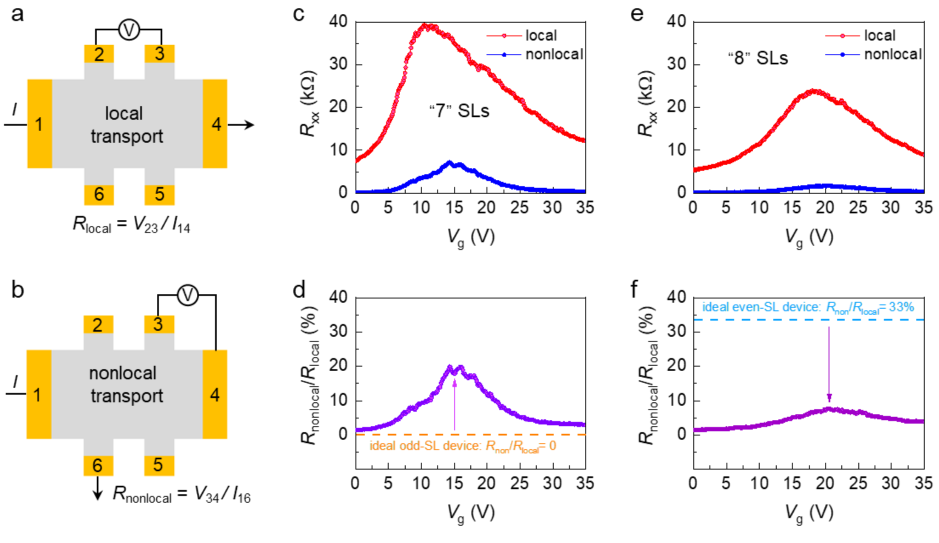

Fig. S11 | V_g-dependent local and nonlocal transport behaviors in devices S6 and S7. a,

b, Configurations of the local (**a**) and nonlocal (**b**) measurements in a six-terminal Hall bar structure. **c,e,** Local and nonlocal resistance of "7"-SL (**c**) and "8"-SL (**e**) device as a function of V_g. **d,f,** V_g-dependent $R_{nonlocal}/R_{local}$ of "7"-SL (**d**) and "8"-SL (**f**) devices.

Reference

1. Gao A., Liu Y. F., Hu C.*, et al.* Layer Hall effect in a 2D topological axion antiferromagnet. *Nature*, **595**, 521-525 (2021).
2. Zhu J., Feng Y., Zhou X.*, et al.* Direct observation of chiral edge current at zero magnetic field in odd-layer $MnBi_2Te_4$. p. arXiv:2307.10150; 2023.
3. Liu C., Wang Y., Li H.*, et al.* Robust axion insulator and Chern insulator phases in a two-dimensional antiferromagnetic topological insulator. *Nat. Mater.*, **19**, 522-527 (2020).
4. Li Y., Liu C., Wang Y., Lian Z., Li H., Wu Y., Zhang J., Wang Y. Nonlocal Transport and One-dimensional Conduction in the Axion Insulator State of $MnBi_2Te_4$. *arXiv*, 2105.10390 (2021).
5. Chen R., Li S., Sun H. P., Liu Q. H., Zhao Y., Lu H. Z., Xie X. C. Using nonlocal surface transport to identify the axion insulator. *Phys. Rev. B*, **103**, L241409 (2021).
6. Jiang S. W., Li L. Z., Wang Z. F., Mak K. F., Shan J. Controlling magnetism in 2D CrI_3 by electrostatic doping. *Nat. Nanotechnol.*, **13**, 549-+ (2018).
7. Gibertini M., Koperski M., Morpurgo A. F., Novoselov K. S. Magnetic 2D materials and heterostructures. *Nat. Nanotechnol.*, **14**, 408-419 (2019).
8. Yan J. Q., Zhang Q., Heitmann T.*, et al.* Crystal growth and magnetic structure of $MnBi_2Te_4$. *Phys. Rev. Mater.*, **3**, 064202 (2019).
9. Ding L., Hu C. W., Ye F., Feng E. X., Ni N., Cao H. B. Crystal and magnetic structures of magnetic topological insulators $MnBi_2Te_4$ and $MnBi_4Te_7$. *Phys. Rev. B*, **101**, 020412(R) (2020).
10. Yang S. Q., Xu X. L., Zhu Y. Z.*, et al.* Odd-Even Layer-Number Effect and Layer-Dependent Magnetic Phase Diagrams in $MnBi_2Te_4$. *Phys. Rev. X*, **11**, 011003 (2021).
11. Bartram F. M., Leng Y. C., Wang Y. C.*, et al.* Ultrafast coherent interlayer phonon dynamics in atomically thin layers of $MnBi_2Te_4$. *Npj Quantum Mater*, **7**, 84 (2022).
12. Mazza A. R., Lapano J., Meyer H. M.*, et al.* Surface-Driven Evolution of the Anomalous Hall Effect in Magnetic Topological Insulator $MnBi_2Te_4$ Thin Films. *Adv Funct Mater*, **32**, 2202234 (2022).
13. Tay H., Zhao Y.-F., Zhou L.-J., Zhang R., Yan Z.-J., Zhuo D., Chan M. H. W., Chang C.-Z. Environmental Doping-Induced Degradation of the Quantum Anomalous Hall

Insulators. *Nano Lett*, **23**, 1093-1099 (2023).

14. Akhgar G., Li Q. L., Di Bernardo I.*, et al.* Formation of a Stable Surface Oxide in $MnBi_2Te_4$ Thin Films. *Acs Appl Mater Inter*, **14**, 6102-6108 (2022).

15. Gao Z., Guo M., Lian Z.*, et al.* Low-damage photolithography for magnetically doped $(Bi,Sb)_2Te_3$ quantum anomalous Hall thin films. *Chin. Phys. B*, **32**, 117303 (2023).
