# Supplementary material for: Fabrication-induced even-odd discrepancy of magnetotransport in few-layer MnBi2Te4
Source: Nat Commun. 2024 Apr 22;15:3399. doi: 10.1038/s41467-024-47779-3 (PMC11035656; doi:10.1038/s41467-024-47779-3)
Supplement: Supplementary file 2 — Peer Review File [file 41467_2024_47779_MOESM2_ESM.pdf]

nature portfolio

Peer Review File

Fabrication-induced even-odd discrepancy of magnetotransport in few-layer MnBi2Te4

Open Access This file is licensed under a Creative Commons Attribution 4.0 International License, which permits use, sharing, adaptation, distribution and reproduction in any medium or format, as long as you give appropriate credit to the original author(s) and the source, provide a link to the Creative Commons license, and indicate if changes were made. In the cases where the authors are anonymous, such as is the case for the reports of anonymous peer reviewers, author attribution should be to 'Anonymous Referee' followed by a clear attribution to the source work. The images or other third party material in this file are included in the article's Creative Commons license, unless indicated otherwise in a credit line to the material. If material is not included in the article's Creative Commons license and your intended use is not permitted by statutory regulation or exceeds the permitted use, you will need to obtain permission directly from the copyright holder. To view a copy of this license, visit http://creativecommons.org/licenses/by/4.0/.

REVIEWER COMMENTS

Reviewer #1 (Remarks to the Author):

The manuscript reports the magnetotransport properties of MnBi2Te4 exfoliated flakes and their optical contrasts. The authors observed zero Hall resistivity in the magnetic field dependence in both 6 septuple layer (SL) and 7 SL devices and found distinct differences in their robustness against the gate voltage. The authors also investigated the optical contrasts of the flakes under the microscope and found the contrasts changed after the device fabrication. The authors interpreted the contrast changes by the reduction of the effective layer thickness and discussed that the reduced effective thickness can explain the observation of zero Hall resistivity in the 7 SL device. The research was conducted systematically, and the conclusion of the paper sounds comprehensive. I believe that the content of this paper is quite useful in sorting out the confusion in the experimental studies of MnBi2Te4 exfoliated flakes. Therefore, I agree to publish the manuscript basically. But, I have several concerns about the manuscript as described below and would ask the authors to address them.

1) In lines 90-93, the authors claim “We find the existence of two distinct zero Hall phenomena: the robust zero Hall plateau state in a 6-SL MnBi2Te4 corresponding to the axion insulator state expected for even-number-SL devices, and an accidental zero Hall plateau in a 7-SL device but with reduced Oc after fabrication”. I suspect that the two different appearances of the zero Hall resistivity plateau are due to the quantitative difference in the energy gap, but not a qualitative one. If this is the case, I am reluctant to call them “two distinct zero Hall phenomena” or “two types of zero Hall phenomena”. Actually, the magnetic field dependences of \rho_{yx} and \rho_{xx} for the 6-SL device at T = 12 K look very similar to those for the 7-SL device at T = 1.5K. If the experiment was conducted at lower temperatures, a zero plateau might appear in the gate voltage dependence of d\rho_{yx} /dH even in the 7-SL device. In my opinion, these are not two different phenomena but two different appearances or manifestations of the zero Hall resistivity phenomena associated with the axion insulator with different energy gaps. If the author agrees with my opinion, please correct the wording. If not, please add data and discussions to show that the two zero Hall resistivity plateaus are different qualitatively.

2) In this study, the authors used four different crystals grown by four different growth methods. Please specify the crystals and the growth methods used for fabricating the 6-SL and 7-SL devices (S2 and S6).

3) If S2 and S6 are fabricated from the same crystal, why Oc did not change in S2 but in S6? Is this just a coincidence?

4) In lines 200-201, the authors state "that was rarely noticed in previous studies of 2D materials". I think this statement implies that PMMA does not affect the other 2D materials severely. I would like to know why MnBi2Te4 is especially weak to PMMA compared to the other 2D materials.

5) The error bars in Fig. 1d are hardly visible. Please make them clear. To make the meaning of the error bars clear please provide information about how many samples were measured for each thickness.

6) What was the magnetic field for the measurement in Fig. 1e?

7) Please provide the information on the measurement temperature in the figure captions of Figs. 2 and 3.

8) In Fig. 4b, some star symbols are plotted. What do they stand for? Please provide an explanation in the caption.

9) In Figs. 4f and 4g, the data for the devices S1-S7 are provided. Were they fabricated from the same crystal? Please specify the crystals of these devices.

10) In Fig. 4e, the authors illustrate the band-structure-like parabolas with an intentionally narrowed band gap for the top surface. However, no explanation is provided for this band-structure-like cartoon. I cannot understand why the gap becomes narrow after the fabrication. Please elaborate more on this cartoon.

Reviewer #2 (Remarks to the Author):

Li and coworkers performed systematic optical and transport studies of exfoliated few layers flakes of MnBi2Te4 devices. The authors surveyed the optical contrast (Oc) in over 200 MnBi2Te4 flakes. The statistical results suggest that the reduction of Oc after PMMA covering correlates with sample sources. The magneto-transport studies of seven representative devices suggest the reduction of Oc correlates with effective reduction of 1 septuple layer (SL) of MnBi2Te4. The presented results are timely and important for the 2D community, especially for the researchers working on MnBi2Te4 and related topological materials. However, I did not find the evidence convincing. In particular, the calibration of flake thickness is susceptible to extrinsic effects. Furthermore, the authors did not provide a satisfactory mechanism of the sample dependence of the response to fabrication (PMMA covering), which is crucially important for the 2D community to avoid the fabrication issue. Therefore, I cannot recommend publishing the manuscript in the present form. Below are my comments.

1. The motivation of this work is to address the conflicting observations of anomalous Hall effect and magnetization in flakes with different thicknesses, especially the conflicting results from different groups. Therefore, it would be very informative to perform these characterizations (e.g., transport and magneto-optics measurements) on the same device because AFM characterization of flake thickness could be susceptible to various extrinsic effects.

2. The explanation of reverse chirality of anomalous Hall loop is confusing. The authors speculate that broken inversion symmetry could result in opposite net magnetization of degraded even-layer flake after reducing magnetic field. I cannot follow the logic because broken inversion symmetry doesn’t couple to magnetization. How can the system choose a state with higher Zeeman energy? Again, it would be very informative to measure the sample magnetization of the same device.

Reviewer #3 (Remarks to the Author):

The antiferromagnetic (AFM) topological insulator (TI) MnBi2Te4 holds promising potential for investigating novel topological phenomena such as quantum anomalous Hall and axion insulator. However, the fabricated device's electronic structure may undergo substantial alterations, giving rise to controversies regarding the topological nature of MnBi2Te4 in its ground state. To this end, the authors aimed to elucidate the influence of fabrication on MnBi2Te4. In this study, the authors demonstrate the existence of two distinct types of zero Hall phenomena in few-layer MnBi2Te4. They conduct a statistical survey of the optical contrast in more than 200 MnBi2Te4 samples, revealing that the accidental zero Hall phenomenon may arise from the reduction of effective thickness during the fabrication process. Overall, the paper is well-structured and accessible, and the results are easy to comprehend. However, I believe the most valuable aspect of the paper is not the two types of zero Hall phenomena. Instead, it accentuates critical concerns related to the fabrication and characterization of devices based on 2D materials.

There are several points which should be addressed by the authors.

1.The authors claim to have discovered the presence of two distinct zero Hall phenomena. One is derived from a 6-SL MnBi2Te4, corresponding to the axion insulator state, while the other originates from a 7-SL MnBi2Te4 device. However, they attribute the zero Hall plateau observed in the 7-SL device to a reduction of 1 SL following the commonly adopted fabrication method for 2D materials. If this indeed holds true, labeling these as two separate zero Hall phenomena appears misleading. According to the authors' findings, the 7-SL MnBi2Te4 device effectively transforms into a 6-SL MnBi2Te4 device. Consequently, both devices should be classified as constituents of the axion insulator state.

2. The paper's primary advantage lies in its discovery that the fabrication of devices based on 2D materials can modify the MnBi2Te4 ground state. How widespread is this issue within MnBi2Te4 fabrications? Does it occur in most MnBi2Te4 fabrications or is it specific to the samples discussed in this paper?

3. In Fig. 1c, the Oc appears to increase linearly with the SL thickness. However, in Fig. 4c, the Ocs of certain MnBi2Te4 samples in crystal #1 increase after fabrication. Does this suggest an increase in the effective layer thickness?

4. The images presented in Fig. 4(d-e) appear to bear resemblance to the first-principles calculations outlined in arXiv:2205.08204. It would provide valuable insights if the authors could juxtapose their findings with the results of the first-principles calculations or model calculations from arXiv:2205.08204."

In this letter we provide a point-to-point response to the reviewers' comments.

In the following, the reviewers' original comments are shown by blue italic characters.

The original sentences in the reference are indicated by red italic characters.

The authors' responses are shown by black normal characters.

Reviewer #1:

The manuscript reports the magnetotransport properties of $MnBi_2Te_4$ exfoliated flakes and their optical contrasts. The authors observed zero Hall resistivity in the magnetic field dependence in both 6 septuple layer (SL) and 7 SL devices and found distinct differences in their robustness against the gate voltage. The authors also investigated the optical contrasts of the flakes under the microscope and found the contrasts changed after the device fabrication. The authors interpreted the contrast changes by the reduction of the effective layer thickness and discussed that the reduced effective thickness can explain the observation of zero Hall resistivity in the 7 SL device. The research was conducted systematically, and the conclusion of the paper sounds comprehensive. I believe that the content of this paper is quite useful in sorting out the confusion in the experimental studies of $MnBi_2Te_4$ exfoliated flakes. Therefore, I agree to publish the manuscript basically. But I have several concerns about the manuscript as described below and would ask the authors to address them.

We thank the reviewer for the nice summary and the favorable comments of our work.

1. In lines 90-93, the authors claim "We find the existence of two distinct zero Hall phenomena: the robust zero Hall plateau state in a 6-SL $MnBi_2Te_4$ corresponding to the axion insulator state expected for even-number-SL devices, and an accidental zero Hall plateau in a 7-SL device but with reduced O_c after fabrication". I suspect that the two different appearances of the zero Hall resistivity plateau are due to the quantitative difference in the energy gap, but not a qualitative one.

If this is the case, I am reluctant to call them "two distinct zero Hall phenomena" or "two types of zero Hall phenomena". Actually, the magnetic field dependences of ρ_{yx} and ρ_{xx} for the 6-SL device at T = 12 K look very similar to those for the 7-SL device at T = 1.5K. If the experiment was conducted at lower temperatures, a zero plateau might appear in the gate voltage dependence of $d\rho_{yx}/dH$ even in the 7-SL device. In my opinion, these are not two different phenomena but two different appearances or manifestations of the zero Hall resistivity phenomena associated with the axion insulator with different energy gaps. If the author agrees with my opinion, please correct the wording. If not, please add data and discussions to show that the two zero Hall resistivity plateaus are different qualitatively.

We thank the reviewer for pointing out the issue regarding the wording of the two distinct zero Hall effects. After reading the reviewer's comments, we realized that the original descriptions were inaccurate. Following the reviewer's suggestion, we corrected the wordings and described them as "*different manifestations of the zero Hall plateau*". A few sentences on their quantitative differences were added in the revised manuscript.

2. In this study, the authors used four different crystals grown by four different growth methods. Please specify the crystals and the growth methods used for fabricating the 6-SL and 7-SL devices (S2 and S6).

We apologize for not clarifying this issue. Both the 6-SL and 7-SL devices were derived from crystal #1, which was grown by directly mixing Bi_2Te_3 and MnTe with the ratio of 1:1 in a vacuum-sealed silica ampoule. The details about the sample growth were documented in the method section. In the revised manuscript, we added a few sentences to specify the crystal numbers and their growth methods for different devices.

3. If S2 and S6 are fabricated from the same crystal, why O_c did not change in S2 but in S6? Is this just a coincidence?

The reviewer raised a very interesting question. In fact, in the experimental study of $MnBi_2Te_4$, the observation of sample-dependent behaviors is a widespread phenomenon. Whether in transport or spectroscopic studies, it has been found that different crystals, and even different flakes cleaved from the same crystal, may manifest different behaviors [*Science* 367, 895-900 (2020) & *PRB* 104, 115168 (2021) & *npj Quantum Materials* 7, 7 (2022)]. One prevailing understanding attributes this to the various structural defects and non-uniformity in $MnBi_2Te_4$. In our experiments, we attributed the mechanism of the O_c change to the fabrication-catalyzed instability of $MnBi_2Te_4$ surface, which was found to be closely associated with the defect in $MnBi_2Te_4$, as evidenced by TEM measurement and theoretical calculations [*Acs Nano*, 14, 11262 (2020) & *arXiv*: 2205.08204 & *PRL* 130, 126702 (2023)]. Previous experiments combining STM, ARPES and DFT calculation clearly indicated that "*the defects concentration is nonuniform within crystals and differs from sample to sample*" [*PRB* 104, 115168 (2021) & *npj Quantum Materials* 7, 7 (2022)]. Therefore, the different performances in S2 and S6 are likely attributable to the inherent non-uniformity in the $MnBi_2Te_4$ crystals. This is also one of the reasons that we conducted a statistical survey of the O_c variation in over 200 flakes derived from four different crystals, which allows us to capture a wide range of defect distribution. The sample-dependent statistical results reinforce our conclusions regarding the non-uniformity in $MnBi_2Te_4$ crystals and its impacts on device performance.

In the revised manuscript, we added a few words on the reasons for the different performances in devices S2 and S6.

4. In lines 200-201, the authors state "that was rarely noticed in previous studies of 2D materials". I think this statement implies that PMMA does not affect the other 2D materials severely. I would like to know why $MnBi_2Te_4$ is especially weak to PMMA compared to the other 2D materials.

We thank the reviewer for raising this good point. The reason that we can observe the impacts of PMMA is primarily because the ground state properties of $MnBi_2Te_4$ are sensitive to the parity of layer number. Specifically, in odd-number-SL $MnBi_2Te_4$, it shows an anomalous Hall effect with a hysteresis loop at zero magnetic field, while in even-number-SL $MnBi_2Te_4$, it displays a zero Hall plateau with vanished hysteresis. It is important to note that in most other 2D materials, decreasing the effective thickness by one layer typically leads to quantitative change on the transport properties instead of leading to pronounced layer-dependent oscillation. Therefore, while PMMA may indeed affect the transport properties of 2D materials, detecting the O_c variation and its relation to transport may not be readily achievable.

Regarding the weakness of $MnBi_2Te_4$ to PMMA, we believe it is closely related to the defects and the fragility of the $MnBi_2Te_4$ surface. Previous studies have highlighted the substantial impacts of the ubiquitous defects (such as Mn at Bi sites, Bi at Mn sites, Bi at Te sites, and Te vacancy) on the stability of $MnBi_2Te_4$ surface [*Acs Nano*, 14, 11262 (2020) & *Phys. Rev. B*, 104, 115168 (2021)]. For instance, the synergistic effect of a high concentration of Mn-Bi mixings and Te vacancies can trigger a surface collapse and reconstruction process, transforming an ideal SL of $MnBi_2Te_4$ into a QL of Mn-doped Bi_2Te_3 and a double layer of Mn_xBi_yTe. This will naturally give rise to a reduction of effective thickness for $MnBi_2Te_4$ phase by 1-SL. In addition, recent theoretical calculations also suggested that the structural defects can induce a surface charge redistribution in $MnBi_2Te_4$ surface, pushing the surface state from the first SL to the second SL [*PRL* 130, 126702 (2023)]. During the peer-review process, reviewer #3 also raised a relevant work demonstrating that a small expansion of the van der Waals (vdW) gap can result in a noteworthy reduction in the surface band gap [*arXiv*: 2205.08204]. In a (7+1) SL $MnBi_2Te_4$, the Chern number change by 1 naturally gives rise to the 1-SL reduction of the effective thickness for transport (*C* differs by 1 between even- and odd-number-SL $MnBi_2Te_4$). In this work, it was also pointed that "*the van der Waals gap is sensitive to impurities or intercalated atoms in fabrication processes, and even a small concentration of impurities can significantly expand the vdW gap*". Given the substantial defects observed in all available $MnBi_2Te_4$ crystals [*PRX* 11, 021033 (2021) & *ECS Jour. Solid State Sci. Tech*. 11, 063007 (2022) & *PRM* 5,

124206 (2021)], we believe it is the non-uniformity and defects within $MnBi_2Te_4$ that play the most significant role on its weakness to fabrication.

Notably, in the study of 2D martials, it has been observed that PMMA residuals on the sample surface can significantly affect the electrical properties of these materials [*Nano-Micro Lett*, 8, 336-346 (2016) & *APL* 99, 122108 (2011) & *Adv. Mater. Interfaces*, 6, 1801321 (2019)]. It can increase the observed thickness in the AFM measurements through absorption, and serve as a charge source, prompting the charge redistribution process. Our AFM topographical analysis has indeed revealed the presence of island-like PMMA residuals on the surface of on the $MnBi_2Te_4$. In addition, various adsorbates trapped between layers during the fabrication process can also expand the van der Waals gap [*Nat. Mater.*, 11, 764-767 (2012)]. Therefore, it is likely that the combined influences of non-uniformity, defects, and PMMA contribute to sample dependent behaviors during the fabrication.

In the revised manuscript, we added more words in the discussion part about the particularity of $MnBi_2Te_4$ compared to other 2D materials.

5. The error bars in Fig. 1d are hardly visible. Please make them clear. To make the meaning of the error bars clear please provide information about how many samples were measured for each thickness.

Following the reviewer's suggestion, we labeled the quantity of the samples used for thickness determination in the revised Fig. 1d.

6. What was the magnetic field for the measurement in Fig. 1e?

These curves were measured at 0 T. The value of magnetic field was marked in Fig. 1e and its figure caption.

7. Please provide the information on the measurement temperature in the figure captions of Figs. 2 and 3.

These transport measurements were conducted at 1.5 K. The temperature information has been added in the revised figures.

8. In Fig. 4b, some star symbols are plotted. What do they stand for? Please provide an explanation in the caption.

These star symbols represent the data of O_c for the 7 samples displayed in Figs. 4f and 4g. We added a few words in the revised manuscript to clarify it clearly.

9. In Figs. 4f and 4g, the data for the devices S1-S7 are provided. Were they fabricated from the same crystal? Please specify the crystals of these devices.

Devices S1 and S5 were derived from crystal #3 and #2, respectively. All the other five devices were derived from crystal #1. In the revised figures, we specified the crystal source of these devices.

10. In Fig. 4e, the authors illustrate the band-structure-like parabolas with an intentionally narrowed band gap for the top surface. However, no explanation is provided for this band-structure-like cartoon. I cannot understand why the gap becomes narrow after the fabrication. Please elaborate more on this cartoon.

We apologize for not clarifying the schematic cartoons clearly. The narrower band gap can be understood from two aspects. Firstly, the resemblance in the transport behaviors of the 6-SL device at elevated Ts and those of the 7-SL device at lower Ts strongly suggest a reduced gap for the 7-SL device compared to its pristine state. Just as the reviewer commented that the wording of "*distinct zero Hall effects*" in our last manuscript is inaccurate, it is more appropriate to be described as "*two*

different manifestations of zero Hall plateau associated with the axion insulator with different energy gaps."

Secondly, in our manuscript, we attributed the mechanism of the O_c change to the fabrication-catalyzed instability of the $MnBi_2Te_4$ surface. It is naturally to expect a narrower gap in a magnetic TI with perturbated surface. A recent theoretical calculation also highlights that defects can induce a surface charge redistribution and results in a reduced surface gap [*PRL* 130, 126702 (2023)]. The schematic illustration for the relation between charge distribution and surface gap in the *PRL* paper is adapted in Fig. R1. We found this result consistent with our experiment, therefore we depicted a similar cartoon.

In the revised manuscript, we cited these relevant references and added more descriptions on the cartoon pictures.

[FIGURE REDACTED]

Fig. R1. The schematics for the relationship between surface charge distribution and magnetic gap under AFM (a) and FM (b). The figures were adapted from *PRL* 130, 126702 (2023).

Reviewer #2:

Li and coworkers performed systematic optical and transport studies of exfoliated few layers flakes of $MnBi_2Te_4$ devices. The authors surveyed the optical contrast (O_c) in over 200 $MnBi_2Te_4$ flakes. The statistical results suggest that the reduction of O_c after PMMA covering correlates with sample sources. The magneto-transport studies of seven representative devices suggest the reduction of O_c correlates with effective reduction of 1 septuple layer (SL) of $MnBi_2Te_4$. The presented results are timely and important for the 2D community, especially for the researchers working on $MnBi_2Te_4$ and related topological materials. However, I did not find the evidence convincing. In particular, the calibration of flake thickness is susceptible to extrinsic effects. Furthermore, the authors did not provide a satisfactory mechanism of the sample dependence of the response to fabrication (PMMA covering), which is crucially important for the 2D community to avoid the fabrication issue. Therefore, I cannot recommend publishing the manuscript in the present form. Below are my comments.

We thank the reviewer for the nice summary of our work and the favorable comment that our results "*are timely and important for the 2D community, especially for the researchers working on $MnBi_2Te_4$ and related topological materials.*" The reviewer's main concerns are that the calibration of thickness is susceptible to extrinsic effects and we did not provide a satisfactory mechanism of the sample dependence of the response to fabrication, which is important for the 2D community to avoid the fabrication issue.

Firstly, we totally agree with the reviewer that the calibration of thickness could be susceptible to extrinsic effects. We apologize for not clarifying this issue clearly in our last manuscript. In fact, we have checked the thickness of our flakes by different experimental methods, including nonlocal transport, scanning microwave impedance microscopy (MIM), scanning superconducting quantum interference device (SQUID), ultra-fast optical pump-probe reflectivity measurements, and Raman spectroscopy [*Nat. Commun.* 13, 7714 (2022) & *npj Quantum Materials*, 7, 84 (2022) & *Sci. Bull.* 68, 1252 (2023) & *arXiv:* 2307.10150]. All the samples measured in these papers were exfoliated

from the same crystal #1 as used in the current work. In these experiments, these samples displayed consistent layer-dependent properties with the thickness determined by O_c and AFM measurements. Recently, we carried scanning SQUID and MOKE measurements on samples with varied thickness to confirm the thickness calibration and the impact of fabrication on effective thickness. In addition, we also compared the relationship between O_c and thickness obtained in our crystal with the results obtained by another group on different crystals [*Nature* 595, 521 (2021)], and found a high level of consistency. In this *Nature* work, to confirm the accuracy of the thickness calibration by O_c, the thickness was checked by TEM and RMCD measurements. These experiments provide convincing evidence for our experiment. More details are shown in the replies to question 1.

Secondly, regarding the mechanism of the sample dependent responses to fabrication process, we totally agree with the reviewer that it is "*crucially important for the 2D community to avoid the fabrication issue*". In fact, in the experimental study of $MnBi_2Te_4$, it is a widespread phenomenon that $MnBi_2Te_4$ exhibits sample-dependent behaviors. Whether in transport or spectroscopic studies, it has been found that different crystals, or even different thin flakes cleaved from the same crystal, could display different behaviors [*Science* 367, 895-900 (2020) & *PRB* 104, 115168 (2021) & *npj Quantum Materials* 7, 7 (2022)]. One prevailing understanding attributes this to the various defects and non-uniformity of $MnBi_2Te_4$. It was highlighted that the structural defects and perturbation can give rise to the instability of the $MnBi_2Te_4$ surface, for example by inducing a charge redistribution or a van der Waals gap expansion, which can push the surface charge to the second layer or change the Chern number of the top layer [*Acs Nano*, 14, 11262 (2020) & *PRX* 11, 021033 (2021) *& PRL* 130, 126702 (2023) & *arXiv*: 2205. 08204]. Given the intricate physical and chemical processes occurring at the surface in the fabrication process, we attributed the change of O_c to the fabrication-catalyzed instability of $MnBi_2Te_4$ surface. Notably, in previous study of graphene, MoS_2, and WSe_2, it has been observed that the PMMA residuals on the surface can influence the intrinsic electrical properties of the 2D materials [*Nano-Micro Lett*, **8**, 336-346 (2016) & *APL* 99, 122108 (2011) & *Adv. Mater. Interfaces*, 6, 1801321 (2019)]. It can not only increase the observed thickness in the AFM measurement through absorption, but also act as charge source, catalyzing the surface charge

redistribution. Our topography measurement by AFM indeed revealed island-like PMMA residuals on $MnBi_2Te_4$ surface (see supplementary Fig. S8). In addition, various adsorbates trapped between layers during the fabrication process can also expand the van der Waals gap [*Nat. Mater.*, 11, 764-767 (2012)]. Therefore, it is highly likely that the combined influence of non-uniformity, defects, and PMMA contribute to the sample dependent behaviors in response to fabrication.

Although the mechanisms by which PMMA affects the sample quality is not fully understood, in the past few months, we have been devoted to developing new fabrication method to address the issue. Recently, we find that by depositing a protective layer of AlO_x on the $MnBi_2Te_4$ surface prior to spin-coating PMMA, we can fabricate few-layer $MnBi_2Te_4$ devices without O_c reduction. In Fig. R2(a), we present our preliminary results measured on 22 flakes derived from the same crystal (#5). For the 12 flakes without capping layer, all of them exhibit pronounced O_c changes after fabrication. However, for the other 10 flakes with AlO_x capping layer, their O_c remain unchanged. These results indicate that the AlO_x capping layer can protect the sample from damages caused by fabrication to a large extent. Figure R2(b) displays the transport result of a 7-SL device with AlO_x capping layer. The anomalous Hall resistivity 0.1 h/e^2 at zero magnetic field has reached the highest compared to our previous results. Currently, we only obtained some preliminary results. More thorough studies are needed to refine the fabrication process.

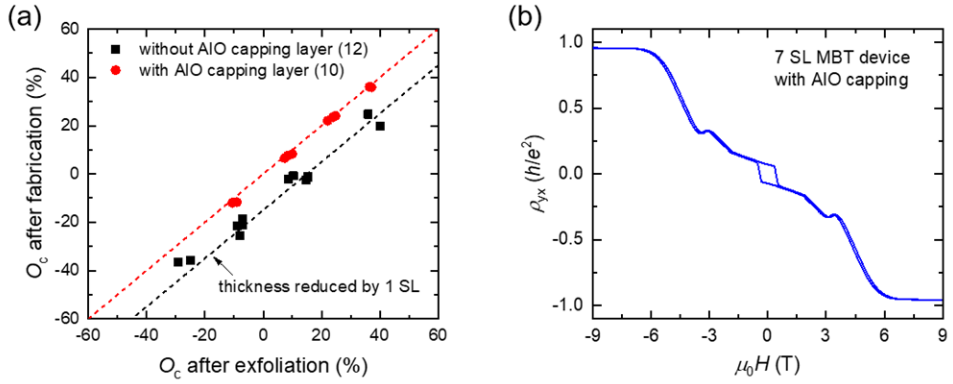

Fig. R2. (a) Summary of the O_c changes for $MnBi_2Te_4$ flakes with and without AlO_x. (b) Magnetic field dependent ρ_{yx} in a 7-SL device with AlO_x capping layer.

In consideration of the reviewer's highly insightful suggestions, in the revised manuscript, we added some descriptions on the consistent behaviors across different experiments. In the discussion paragraph, we cited the relevant references and reorganized the explanations to the fabrication issue. We also included our new scanning SQUID and MOKE results in the supplementary information. In addition, our modified fabrication method and the preliminary optical and transport results were added in the supplementary information. We believe these results provide compelling evidence for our experiment. We hope that the revised manuscript meets the standard for publication in *Nature Communications*.

1. The motivation of this work is to address the conflicting observations of anomalous Hall effect and magnetization in flakes with different thicknesses, especially the conflicting results from different groups. Therefore, it would be very informative to perform these characterizations (e.g., transport and magneto-optics measurements) on the same device because AFM characterization of flake thickness could be susceptible to various extrinsic effects.

The most significant finding in our experiment lies in the revelation of the fabrication-induced even-odd discrepancy of magnetotransport in few-layer $MnBi_2Te_4$. We concur with the reviewer's insight that conducting different measurements on the same device is very informative, particularly considering the susceptibility of AFM characterizations to some extrinsic effects. We apologize for not addressing this issue in our manuscript. In fact, we have extensively investigated the thickness dependent behaviors of few-layer $MnBi_2Te_4$ using various methods. All these samples manifested consistent behaviors with their thicknesses determined by O_c and AFM. Below we list some of the results.

Transport. Figures R3(a) and R3(b) display the standard four-probe transport [Fig. 4(f) in the current work] and the nonlocal transport results [*Sci. Bull.* 68, 1252 (2023)] in few-layer $MnBi_2Te_4$ flakes exfoliated from crystal #1. Theoretically, even-number-layer $MnBi_2Te_4$ is expected to exhibit zero Hall plateau and giant nonlocal resistivity near zero magnetic field, whereas odd-number-layer

$MnBi_2Te_4$ will exhibits anomalous Hall hysteresis and vanished nonlocal signal. If the layer number determined by O_c and AFM has an error of one layer, one will observe a completely opposite trend of transport behavior in relation to the odd-even oscillations with thickness.

Optical contrast, AFM, time-resolved reflectivity, and polarized Raman spectroscopy. In Figs. R3(c) and R3(d), we show the O_c and AFM result measured independently by our collaborator in a different AFM facility. The relationship between O_c and thickness is highly consistent with the result in current experiment. In the same flakes, time-resolved reflectivity and Raman spectroscopy measurement were also performed. All the measurements revealed consistent thickness dependent oscillation frequency and Raman shift [Figs R3(e) and R3(f)] with the thickness determined by O_c and AFM, which undoubtedly suggest that the calibration of thickness in our work is accurate [*npj Quantum Materials*, 7, 84 (2022)].

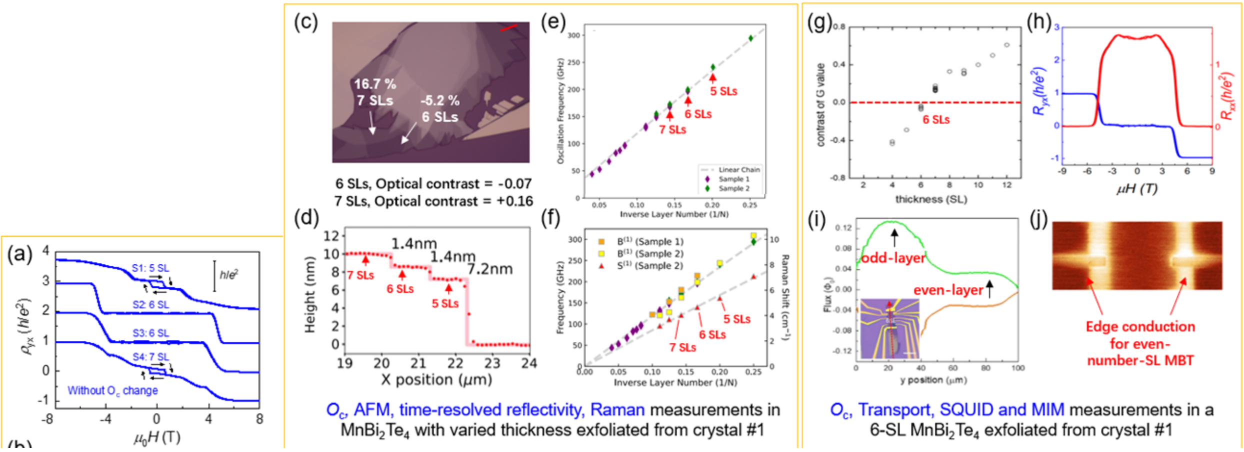

[Panel B has been redacted]

Fig. R3. (a)-(b) Thickness dependence of four-probe and nonlocal transport behaviors of few-layer $MnBi_2Te_4$ exfoliated from crystal #1. (b) is adapted from *Sci. Bull.* 68, 1252 (2023). (c)-(d) Optical image and AFM measurements of few-layer $MnBi_2Te_4$ exfoliated from crystal #1. (e)-(f) Thickness dependent oscillation frequency and Raman shift obtained by time-resolved reflectivity and Raman spectroscopy measurements in $MnBi_2Te_4$ flakes exfoliated from crystal #1. (c)-(f) are adapted from *npj Quantum Materials*, 7, 84 (2022). (g) Relation between O_c and thickness for $MnBi_2Te_4$ flakes exfoliated from crystal #1. (h) Magnetic field dependent transport properties for a 6-SL $MnBi_2Te_4$.

(i) Flux line-cut profiles across the 6-SL and the thicker region for the $MnBi_2Te_4$ device. Different colors denote the result acquired after a positive and negative initial magnetization. (j) Visualization of the edge conduction in a 6-SL $MnBi_2Te_4$. (g)-(j) are adapted from *Nat. Commun.* 13, 7714 (2022).

Optical contrast, Transport, SQUID and MIM. In addition to the O_c and transport, we also performed scanning SQUID and scanning MIM measurements on a 6-SL $MnBi_2Te_4$ [*Nat. Commun.* 13, 7714 (2022)]. Figures R3(h) and R3(j) display the transport behaviors and the visualized edge conduction channel that is expected for even-number-layer $MnBi_2Te_4$. To check the even-odd layer property, we further measured the magnetization of the sample by a scanning SQUID, as shown in Fig. R3(i). It clearly shows that the magnetic flux signal in the 6-SL area is only 25 % of that in the thicker area, justifying the even-layer (odd-layer) assignment to the 6-SL region (thicker area). The small net magnetization in the 6-SL part is likely to originate from extrinsic effects, such as frequent thermal cycling, transfer between different systems, and the aging effect.

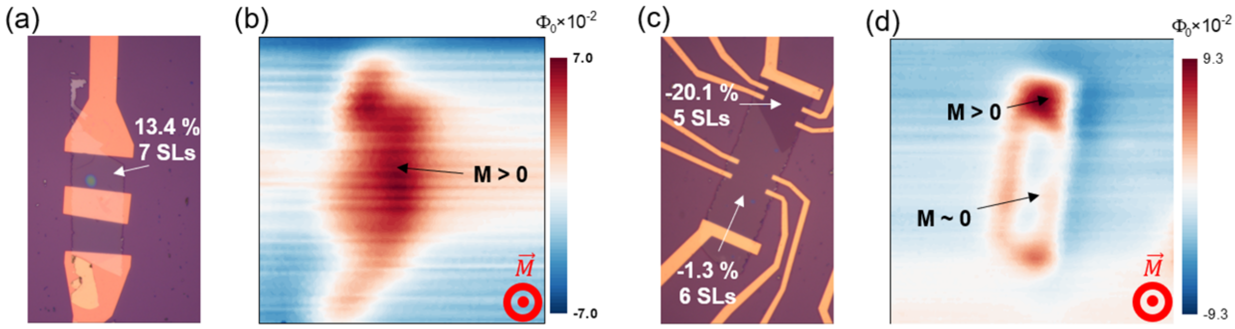

Fig. R4. (a) Optical image of a 7-SL $MnBi_2Te_4$. (b) DC magnetic flux imaging of the 7-SL sample by scanning SQUID as $\mu_0 H$ was swept back to zero from positive magnetization. (c) Optical image of a $MnBi_2Te_4$ device with two different thicknesses. (d) DC magnetic flux imaging of the sample across different parts. (d) is adapted from *arXiv*: 2307.10150.

The scanning SQUID method is highly valuable for checking the calibration of thickness for 2D magnets. The new results obtained in two samples exfoliated from crystal #1 were included in the revised supplementary information. Figure R4 shows the main results for the two samples. For the sample with O_c ~ 13.4 %, its thickness corresponds to ~ 7 SLs. In alignment with expectations,

the DC magnetic flux imaging revealed a pronounced magnetization when $\mu_0 H$ was swept to zero from positive as shown in Fig. R4(b). For the second device, due to the different thickness across the sample, the contrast of the optical properties and the DC flux are much clearer. In the area with O_c around -20.1 %, which corresponds to a thickness of 5 SLs, a pronounced magnetization was observed in the scanning SQUID measurement. Conversely, in the region with O_c of -1.3 %, which corresponds to a thickness of 6 SLs, the magnetization signal is strongly suppressed. These results further demonstrate the accuracy of our thickness calibration. More detailed information about the results of scanning SQUID of $MnBi_2Te_4$ samples can be refereed to *arXiv*:2307.10150.

Optical contrast and MOKE. In the revised manuscript, we also included the results of polar MOKE measurement on two devices with varied thickness. By comparing the MOKE variation in the same sample before and after contact with PMMA, we provide further evidence for the impacts of fabrication on the effective thickness. As presented in Fig. R5(a), for the sample with an initial O_c of 12.8 %, the thickness corresponds to 7 SLs according to the relation between O_c and thickness. This value was confirmed by the measurement of coherent interlayer phonon frequency. As shown in Fig. R5(b), the coherent interlayer phonon frequency was measured to be around ~ 184 GHz, in line with the value for 7-SL $MnBi_2Te_4$ measured in our previous studies [*npj Quantum Materials* 7, 84 (2022)]. After contact with PMMA, the O_c value reduces significantly to 1.8 %, suggesting a reduction in its effective thickness. Correspondingly, a weakening of magnetization was observed, as indicated by the arrow in Fig. R5(c). The different variations in Kerr rotation and Kerr ellipticity mainly result from the different responses of the real and imaginary parts of the optical conductivity to the probe wavelength. However, we noted that the phonon frequency exhibits negligible change after PMMA contact, which demonstrates that the physical thickness remains essentially unaltered. Similar results were reproduced in another sample with different thickness (5 SLs) [Figs. R5(d) to R5(f)]. Notably, the entire measurements were conducted without a protective layer on the sample, hence, we cannot avoid the potential influence of other extrinsic factors on the sample quality, such as environmental doping, aging effect, and surface oxidation. We indeed observed that the initial MOKE signals for both two samples exhibit characteristics deviating from ideal odd-number-SL

$MnBi_2Te_4$, which indicate potential damage from extrinsic effects during the exfoliation or transfer processes. Nevertheless, these consistent results provide compelling evidence for our experiment, validating our thickness calibration and demonstrating the influence of the fabrication on effective thickness.

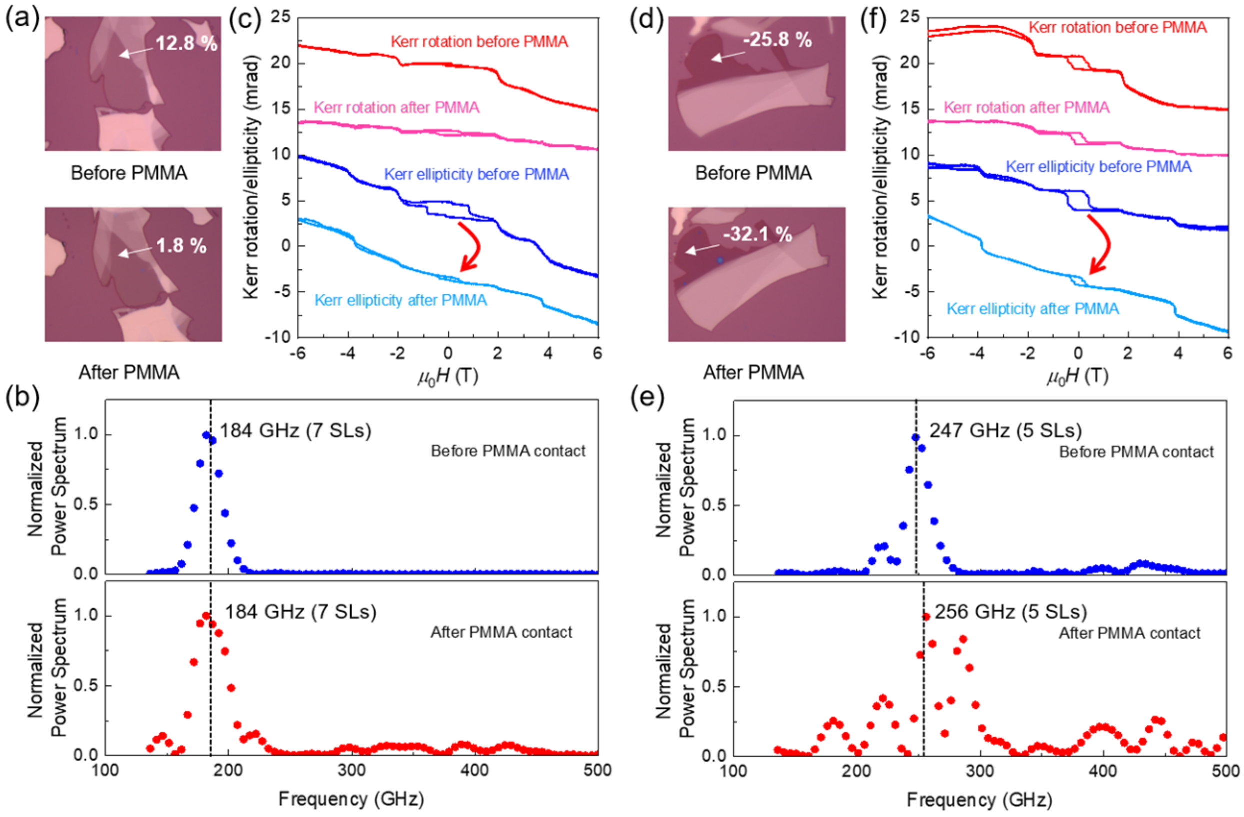

Fig. R5. (a)-(c) Optical images, MOKE, and phonon frequency results for a 7-SL $MnBi_2Te_4$ before and after PMMA contact. (d)-(f) Optical images, MOKE, and phonon frequency results for a 5-SL $MnBi_2Te_4$ before and after PMMA contact.

In addition to these consistent results obtained in our crystal, we find good agreement between our result and that measured independently by another group in different crystals [*Nature* 595, 521 (2021)]. In this *Nature* paper, the thickness was double-checked by TEM and RMCD measurements. Figures R6(a) and R6(b) show the one-to-one correspondence between O_c and thickness measured in our experiments and that in the *Nature* work. In both results, O_c changes from negative to positive when thickness increases from 6 SLs to 7 SLs. The twofold disparity in the absolute values of O_c

arises from the inclusion of the intensity of flake (I_{flake}) in the denominators of the O_c formulas, as marked on top of the figures. The cross-section TEM image for a 6-SL device clearly indicates that the thickness determined by O_c is accurate [inset of Fig. R6(b)]. Figures R6(c) and R6(d) show the RMCD results for three $MnBi_2Te_4$ with varied thicknesses. Nonzero RMCD signal is only observed in the 5-SL and 7-SL areas. While the 6-SL part displays no observable RMCD signal.

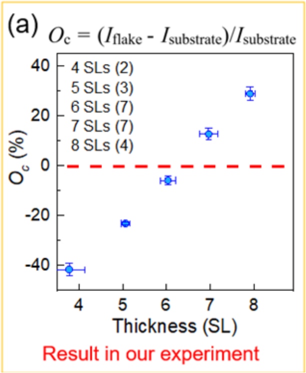

[PANELS B-D ARE REDACTED]

Fig. R6. (a)-(b) Relations between optical contrast and layer number of $MnBi_2Te_4$ flakes measured in our experiment (a) and in the *Nature* paper (b). In our work, the optical contrast O_c is defined as $(I_{flake} - I_{substrate})/I_{substrate}$, where I_{flake} and $I_{substrate}$ are the intensity of $MnBi_2Te_4$ flake and substrate. In the *Nature* paper, it is defined as $(I_{flake} - I_{substrate})/ (I_{flake} + I_{substrate})$. The inset in (b) displays a cross-section TEM image of a 6-SL $MnBi_2Te_4$. (c)-(d) RMCD measurements on $MnBi_2Te_4$ with different thickness. (b)-(d) are adapted from *Nature* 595,521 (2021).

Finally, we would like to point that we concur with the reviewer that performing transport and magneto-optic measurements on the same device is informative. However, it should be highlighted that transferring $MnBi_2Te_4$ between different systems while ensuring the sample quality unchanged is challenging. Frequent thermal cycling, environmental doping, aging effect, and other factors may introduce extrinsic effects on the transport and magnetic behaviors [*Adv. Funct. Mater*. 32, 2202234 (2022) & *Nano Lett.* 23, 1093 (2023)]. This is also why researches typically measured the thickness in a glovebox before performing transport and magnetic measurements. We have been developing more effective method for protecting the sample and look forward to conducting such measurement in the future. Nonetheless, the consistent behaviors between O_c and AFM, O_c and transport, O_c and

phonon dynamics, O_c and scanning MIM, O_c and scanning SQUID, O_c and MOKE, and the results obtained by different group, collectively provide compelling evidence for our experiments.

In the revised manuscript, we added some descriptions on these consistent results and included the scanning SQUID and MOKE results in the revised supplementary information.

2. The explanation of reverse chirality of anomalous Hall loop is confusing. The authors speculate that broken inversion symmetry could result in opposite net magnetization of degraded even-layer flake after reducing magnetic field. I cannot follow the logic because broken inversion symmetry doesn't couple to magnetization. How can the system choose a state with higher Zeeman energy? Again, it would be very informative to measure the sample magnetization of the same device.

We apologize for the confusions in our last manuscript. The mechanism that can couple broken inversion symmetry with magnetization is the magnetoelectric effect. A recent calculation suggests that an electric field can control the magnetization of $MnBi_2Te_4$ (*arXiv*: 2303.06204) and give rise to the AHE of different chirality. Specifically, the authors mentioned that "*The inversion symmetry breaking from the substrate and external gates splits these two AFM states* (up-down/down-up)" and "*find the orbital magnetization in even SLs has a linear dependence on the electric field, while it remains a constant under varying electric field in odd SLs.*" Therefore, it is possible that broken inversion symmetry can couple with magnetization.

Despite the magnetoelectric coupling provides a possible explanation to the reversed chirality of the AHE, we realized that the direction of electric field in our experiment remains undetermined. We also noticed that many other factors can result in the chirality change in the AHE, including the competition between intrinsic and extrinsic AHE mechanism (*Nano Letters* 20, 709 (2020)), layer-locked hidden Berry curvature (*Nature* 595,521 (2021)), surface oxidation (*Adv. Funct. Mater*. 32, 2202234 (2022)), impurity phase (*ACS Nano* 17, 19022 (2023)), and orbital magnetization (*arXiv*:

2303.06204). Given that the chirality of the AH effect is not the focus of our current study, to avoid any confusion, we have reduced the discussion on this part.

With the introduction of the modified fabrication method and the scanning SQUID and MOKE results, the readability and completeness of our manuscript are enhanced. We hope that this revised manuscript meets the standard for publication in *Nature Communications*.

Reviewer #3:

The antiferromagnetic (AFM) topological insulator (TI) $MnBi_2Te_4$ holds promising potential for investigating novel topological phenomena such as quantum anomalous Hall and axion insulator. However, the fabricated device's electronic structure may undergo substantial alterations, giving rise to controversies regarding the topological nature of $MnBi_2Te_4$ in its ground state. To this end, the authors aimed to elucidate the influence of fabrication on $MnBi_2Te_4$. In this study, the authors demonstrate the existence of two distinct types of zero Hall phenomena in few-layer $MnBi_2Te_4$. They conduct a statistical survey of the optical contrast in more than 200 $MnBi_2Te_4$ samples, revealing that the accidental zero Hall phenomenon may arise from the reduction of effective thickness during the fabrication process. Overall, the paper is well-structured and accessible, and the results are easy to comprehend. However, I believe the most valuable aspect of the paper is not the two types of zero Hall phenomena. Instead, it accentuates critical concerns related to the fabrication and characterization of devices based on 2D materials.

We thank the reviewer for the nice summary of our work and positive comment that "*Overall, the paper is well-structured and accessible, and the results are easy to comprehend.*". We deeply value the reviewer's insights. Following the reviewer's suggestion, we have incorporated additional contents in the revised manuscript, specifically focusing on the fabrication issue and its influences on the magnetotransport of few-layer $MnBi_2Te_4$. Our latest scanning SQUID and MOKE data were discussed in the revised supplementary information. Moreover, we have also added our preliminary results regarding a modified fabrication method in the revised supplementary information, which is crucially important for the 2D community to avoid the fabrication issue.

There are several points which should be addressed by the authors.

1. The authors claim to have discovered the presence of two distinct zero Hall phenomena. One is derived from a 6-SL $MnBi_2Te_4$, corresponding to the axion insulator state, while the other originates from a 7-SL $MnBi_2Te_4$ device. However, they attribute the zero Hall plateau observed in

the 7-SL device to a reduction of 1 SL following the commonly adopted fabrication method for 2D materials. If this indeed holds true, labeling these as two separate zero Hall phenomena appears misleading. According to the authors' findings, the 7-SL $MnBi_2Te_4$ device effectively transforms into a 6-SL $MnBi_2Te_4$ device. Consequently, both devices should be classified as constituents of the axion insulator state.

We thank the reviewer for the valuable comments. The reviewer is correct that it is misleading to label the zero Hall effects as *distinct phenomena*. Following the suggestions by reviewers #1 and #3, we corrected the wordings and described them as "*two different manifestations of the zero Hall plateau associated with the axion insulator with different energy gap*".

2. *The paper's primary advantage lies in its discovery that the fabrication of devices based on 2D materials can modify the $MnBi_2Te_4$ ground state. How widespread is this issue within $MnBi_2Te_4$ fabrications? Does it occur in most $MnBi_2Te_4$ fabrications or is it specific to the samples discussed in this paper?*

In our research over the past few years, we have observed that the fabrication issue has exerted varying degrees of influence on most $MnBi_2Te_4$ samples. We believe that this effect is widespread rather than specific to some certain samples. Furthermore, it is important to note that the fabrication method in our work is commonly adopted in 2D materials community. To the best of our knowledge, prior studies have not investigated the influence of fabrication on the O_c and transport properties of $MnBi_2Te_4$. However, based on the findings in the published literature, we posit that this issue is prevalent. For example, in the two papers that combine transport and RMCD measurements [*Nano Lett*, 21, 2544 (2021) & *Nat. Commun.*, 13, 1668 (2022)], at least four samples (5, 6, 7, and 8 SLs) demonstrate anomalous correlations between thickness and transport behaviors, as depicted in Fig. R7. Specifically, the 5- and 7-SL samples exhibit magnetic hysteresis loops in the RMCD signals but lack anomalous Hall effects in transport. Conversely, for the 6- and 8-SL device, RMCD signals are nearly absent while the anomalous Hall effects are pronounced. It is noteworthy that all these

transport devices were fabricated using the same EBL method as adopted in our work (with PMMA covering), and the RMCD measurements were conducted prior to transport before contacting with PMMA. The authors explicitly mentioned that "*To study layer dependent magnetic states by RMCD, the flakes presented in Fig. 1 were freshly exfoliated, sealed in a vacuum protected sample mount, and measured without PMMA covering.*". These results strongly suggest that the fabrication issue is widespread in the study of $MnBi_2Te_4$, rather than specific to the samples in our paper.

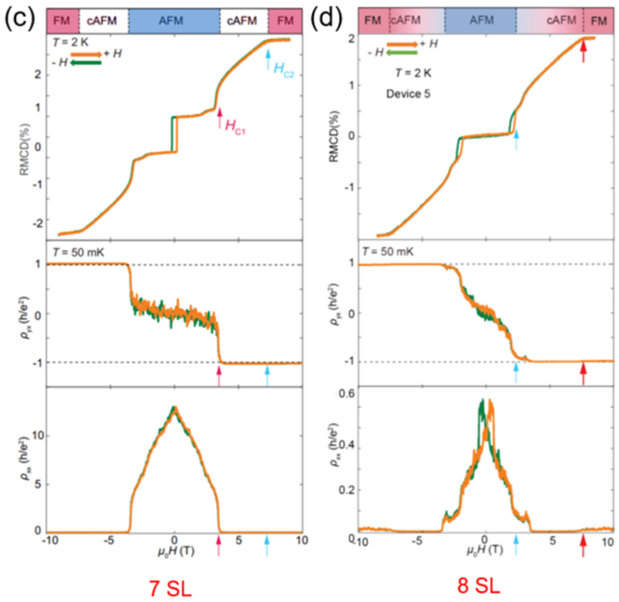

[PANELS A-B ARE REDACTED]

Fig. R7. Correlation of magnetic and topological properties of even and odd SL $MnBi_2Te_4$ flakes. The figures are adapted from *Nano Lett*, 21, 2544 (2021) & *Nat. Commun.*, 13, 1668 (2022).

3. In Fig. 1c, the O_c appears to increase linearly with the SL thickness. However, in Fig. 4c, the O_cs of certain $MnBi_2Te_4$ samples in crystal #1 increase after fabrication. Does this suggest an increase in the effective layer thickness?

We thank the reviewer for raising this interesting point. According to the theorical calculations (*arXiv*: 2205.08204), it is indeed feasible that the effective thickness could increase after fabrication (~ 0.1 Å). However, within the precision of our AFM measurements, we did not observe a notable

increase in the physical thickness after fabrication. The increase of O_c in certain samples is likely a result of measurement errors. From the aspect of experiment, subtle variations in the measurement conditions can lead to experimental errors in O_c, such as the position of the flakes in the light fields, the uniformity of illumination, the size and shape of the sample, and even the presence of electrode. Despite our endeavors to mitigate the experimental errors by averaging the O_c obtained at different positions, eliminating experimental uncertainty remains challenging. Nevertheless, compared with the substantial O_c reduction (up to 20%) attributable to fabrication, such minor experimental errors (up to 4%) do not influence the conclusions drawn from our experiments.

In the revised manuscript, we added a few sentences on the measurement of O_c in the revised methods.

4. The images presented in Fig. 4(d-e) appear to bear resemblance to the first-principles calculations outlined in arXiv:2205.08204. It would provide valuable insights if the authors could juxtapose their findings with the results of the first-principles calculations or model calculations from arXiv:2205.08204."

We thank the reviewer for this highly valuable suggestion. After reading this work, we realized that the results in this paper provide a likely theoretical explanation to our experimental observation. In this paper, the authors found that a small expansion of the interlayer van der Waals gap can lead to a decrease of surface gap. Specifically, the authors calculated the band gap evolution for a (7+1) SL $MnBi_2Te_4$ and found the surface gap closes and reopens. During this process, the Chern number change by $\Delta C = 1$ naturally gives rise to the one-SL reduction of the effective thickness for electron transport (C differs by 1 between even- and odd-number-SL $MnBi_2Te_4$). Such mechanism naturally explains that "*the 7-SL $MnBi_2Te_4$ device effectively transforms into a 6-SL $MnBi_2Te_4$ device*".

In the revised manuscript, we juxtaposed the two findings and added more discussions on the possible explanations to our experiments.

Below is a list of the main changes to the manuscript:

(1) Title: we changed the title to "Fabrication-induced even-odd discrepancy of magnetotransport in few-layer $MnBi_2Te_4$"

(2) Author list: Jinjiang Zhu, Liangyang Liu, Yongqian Wang, Bohan Fu, Shuai Yang, Luyi Yang and Yihua Wang were added to the list. Yayu Wang was no longer the corresponding author.

(3) Abstract: some wordings related to the "distinct zero Hall effect" were revised.

(4) Main text: there are several changes to address the questions by reviewers, as discussed above in the response. The new contents are marked in red.

(5) Figures and captions: in Fig. 1d, we labeled the number of the measured samples. In Figs. 1e, 2a, 2b, 3a, and 3b, the measurement magnetic field and temperature for were added. In Figs. 4f and 4g, the crystal number for different devices were specified.

(6) References: we added refs. 36, 37, 39, 40, 48, 49, 52-54, 56-61 to address the issues raised by the reviewers.

(7) Supplementary information: In section A, we included the results of scanning SQUID on two samples. In section F, we discussed the variation of MOKE signals in two samples before and after PMMA contact. In section G, we added our preliminary result of the modified fabrication method.

REVIEWERS' COMMENTS

Reviewer #1 (Remarks to the Author):

The authors have addressed all my questions and comments. I think the manuscript has improved a lot. I would like to recommend publishing the manuscript in Nature Communications.

Reviewer #3 (Remarks to the Author):

In general, the authors have adeptly addressed my comments, and the paper's conclusion is comprehensive. The study brings attention to crucial concerns regarding the fabrication and characterization of devices employing 2D materials. The results presented in this paper are not only timely but also hold significance for researchers focusing on MnBi2Te4 flakes. I believe that the paper furnishes essential clues to resolve the controversial issue in MnBi2Te4 transport experiments. Consequently, I recommend publishing the manuscript in its current form.

In this letter we provide a point-to-point response to the reviewers' comments.

In the following, the reviewers' original comments are shown by blue italic characters.

The authors' responses are shown by black normal characters.

Reviewer #1:

The authors have addressed all my questions and comments. I think the manuscript has improved a lot. I would like to recommend publishing the manuscript in Nature Communications.

We thank the reviewer for the nice summary and the favorable comments of our work.

Reviewer #2:

In general, the authors have adeptly addressed my comments, and the paper's conclusion is comprehensive. The study brings attention to crucial concerns regarding the fabrication and characterization of devices employing 2D materials. The results presented in this paper are not only timely but also hold significance for researchers focusing on MnBi2Te4 flakes. I believe that the paper furnishes essential clues to resolve the controversial issue in MnBi2Te4 transport experiments. Consequently, I recommend publishing the manuscript in its current form.

We thank the reviewer for the nice summary and the favorable comments of our work.
